# Acute-on-chronic: using magnetic resonance imaging to disentangle the haemodynamic responses to acute and chronic fetal hypoxaemia

**DOI:** 10.3389/fmed.2024.1340012

**Published:** 2024-06-12

**Authors:** Jack R. T. Darby, Brahmdeep S. Saini, Stacey L. Holman, Sarah J. Hammond, Sunthara Rajan Perumal, Christopher K. Macgowan, Mike Seed, Janna L. Morrison

**Affiliations:** ^1^Early Origins of Adult Health Research Group, Health and Biomedical Innovation, UniSA: Clinical and Health Sciences, University of South Australia, Adelaide, SA, Australia; ^2^Peter Gilgan Centre for Research and Learning, The Hospital for Sick Children, Research Institute, Toronto, ON, Canada; ^3^Preclinical, Imaging & Research Laboratories, South Australian Health & Medical Research Institute, Adelaide, SA, Australia; ^4^Department of Physiology, Faculty of Medicine, University of Toronto, Toronto, ON, Canada

**Keywords:** MRI, fetus, hypoxaemia, acute hypoxaemia, chronic hypoxaemia, hypoxia, IUGR, FGR

## Abstract

**Introduction:**

The fetal haemodynamic response to acute episodes of hypoxaemia are well characterised. However, how these responses change when the hypoxaemia becomes more chronic in nature such as that associated with fetal growth restriction (FGR), is less well understood. Herein, we utilised a combination of clinically relevant MRI techniques to comprehensively characterize and differentiate the haemodynamic responses occurring during acute and chronic periods of fetal hypoxaemia.

**Methods:**

Prior to conception, carunclectomy surgery was performed on non-pregnant ewes to induce FGR. At 108–110 days (d) gestational age (GA), pregnant ewes bearing control (*n* = 12) and FGR (*n* = 9) fetuses underwent fetal catheterisation surgery. At 117–119 days GA, ewes underwent MRI sessions where phase-contrast (PC) and T_2_ oximetry were used to measure blood flow and oxygenation, respectively, throughout the fetal circulation during a normoxia and then an acute hypoxia state.

**Results:**

Fetal oxygen delivery (DO_2_) was lower in FGR fetuses than controls during the normoxia state but cerebral DO_2_ remained similar between fetal groups. Acute hypoxia reduced both overall fetal and cerebral DO_2_. FGR increased ductus venosus (DV) and foramen ovale (FO) blood flow during both the normoxia and acute hypoxia states. Pulmonary blood flow (PBF) was lower in FGR fetuses during the normoxia state but similar to controls during the acute hypoxia state when PBF in controls was decreased.

**Conclusion:**

Despite a prevailing level of chronic hypoxaemia, the FGR fetus upregulates the preferential streaming of oxygen-rich blood via the DV-FO pathway to maintain cerebral DO_2_. However, this upregulation is unable to maintain cerebral DO_2_ during further exposure to an acute episode of hypoxaemia. The haemodynamic alterations required at the level of the liver and lung to allow the DV-FO pathway to maintain cerebral DO_2_, may have lasting consequences on hepatic function and pulmonary vascular regulation after birth.

## 1 Introduction

Fetal growth restriction (FGR) occurs in ~10% of pregnancies and represents a significant risk factor for both poor perinatal and neonatal outcomes (1–4). In comparison to the postnatal environment, *in utero* oxygen availability in uncomplicated pregnancies is already limited to the extent that the *in utero* environment has been described as “Everest *in utero*” (5). This relatively hypoxaemic environment is in fact normal for the developing fetus, who even at these low oxygen levels still operates with a significant safety margin of oxygen availability (6, 7) without evidence of a preference toward anaerobic metabolism (8). As such, further impairments to oxygen availability remove this safety margin and increase the risk of poor outcomes.

Fetal hypoxaemia may occur chronically as is the case with placental insufficiency induced FGR or as acute episodes such as during transient umbilical cord occlusions and myometrial contractions (9–14). The haemodynamic response to acute hypoxia is well characterised in fetal sheep (15, 16). Briefly, an initial bradycardia is followed by a rise in mean arterial pressure and peripheral vascular resistance (13, 17, 18). Importantly, this coincides with a phenomenon known as “brain sparing” whereby a redistribution of cardiac output allows for a greater proportion of oxygenated blood to be delivered to the brain (15).

Although less is known about the haemodynamic profile during chronic hypoxaemia, there is evidence that “brain sparing” still occurs. However, rather than a redistribution of cardiac output (19–21), brain sparing during chronic hypoxaemia may result more from the preferential streaming of oxygenated blood from the placenta through the unique shunts of the fetal circulation toward the brain. Moreover, even less is known about the consequences of a second acute episode of hypoxaemia upon a prolonged period of hypoxaemia (acute-on-chronic) such as that which can occur during FGR. However, there is some evidence to suggest that there may be a sensitising effect on the cardiovascular system in response to acute-on-chronic hypoxaemia (22, 23). It is therefore clear that regardless of the cause or duration of the hypoxaemic insult, intrinsic mechanisms are in place to ensure that the brain has the best opportunity for appropriate development. Unfortunately, despite these intrinsic attempts to protect brain growth, the reduced safety margin of oxygenation and the increased risk that the growth restricted fetus has for additional acute complications in late gestation (24) significantly increases the risk of hypoxaemia induced neurodevelopmental deficits (25).

Although there is a haemodynamic attempt to spare brain growth and development in response to hypoxaemia, it is now clear that the presence of brain sparing does not indicate normal cerebral development (25–28). This is particularly highlighted by the fact that over prolonged periods of hypoxaemia, such as those associated with FGR, vascular tone of the cerebral arteries normalizes and instead cerebral metabolism is downregulated (29). As such it is imperative that we better understand how an acute episode of hypoxaemia impacts both cerebral oxygen delivery (DO_2_) and consumption (VO_2_) in a fetus that is already chronically hypoxaemic.

We have recently validated and utilised a combination of the clinically relevant MRI techniques: cine phase-contrast (PC (30–33)) and T_2_ oximetry (34–36), to thoroughly characterise the fetal circulation and better understand how these haemodynamics are influenced by vasoactive agents (37–39) and FGR in sheep (40, 41), as well as congenital heart disease, maternal sleep position and FGR in humans (20, 42–45). Herein, we aimed to apply these techniques to a well-established sheep model of early-onset FGR to determine the impact of a second hit in the form of an episode of acute hypoxia on the haemodynamics of a fetus that is chronically hypoxaemic.

## 2 Materials and methods

### 2.1 Ethical considerations

All experimental protocols were reviewed and approved by the Animal Ethics Committee of the South Australian Health and Medical Research Institute (SAHMRI) and abided by the Australian Code of Practice for the Care and Use of Animals for Scientific Purposes developed by the National Health and Medical Research Council. Ewes from the SAHMRI farm (Burra, South Australia) were housed in an indoor facility with a constant ambient temperature of 20–22°C and a 12 h light/dark cycle. Ewes were housed in individual pens in view of other sheep and had *ad libitum* access to food and water. All investigators understood the ethical principles outlined in Grundy (46) and the principles of the 3Rs, specifically the reduction of the use of animals in research (47).

### 2.2 Carunclectomy surgery and mating

To induce placental restriction (PR) resulting in FGR, ewes (*n* = 9) underwent carunclectomy surgery. Induction of anaesthesia in ewes was via ketamine and diazepam (7 mg/kg IV and 0.3 mg/kg IV, respectively: Lyppards Pty Ltd.). Anaesthesia was maintained with isoflurane 1.5–2% in oxygen (Provet, SA). The uterus was incised, and most caruncles removed, leaving approximately four visible in each horn of the uterus. Analgesia meloxicam; (0.5 mg/kg, subcutaneously) was administered on the day before surgery and 24 h later (48). Antibiotics were administered (525 mg procaine penicillin and 393.75 mg benzathine penicillin: Duplocillin^®^, Lyppards Pty Ltd.) and 250 mg dihydrostreptomycin (Sigma, St Louis, MO, United States) intramuscularly on the day of surgery. Ewes (Control and PR) were mated with a proven ram, with ultrasound to confirm pregnancy at 50–55 days (d) gestational age (GA; term, 150 days).

### 2.3 Fetal catheterization surgery

At 108–110 days GA, singleton bearing Merino ewes (*n* = 21; control, *n* = 12; PR, *n* = 9) underwent surgery as previously described (49, 50). Anaesthesia was induced with intravenous diazepam (0.3 mg/kg) and ketamine (5 mg/kg) and then maintained with isoflurane (1.5–2.5% in 100% oxygen). Vascular catheters were implanted into the maternal jugular vein, fetal femoral vein, femoral artery as well as the amniotic cavity (49, 50). Ewes received an intramuscular injection of antibiotics 3.5 mL of Duplocillin (150 mg/mL procaine penicillin and 112.5 mg/mL benzathine penicillin; Norbrook Laboratories Ltd., Gisborne, Australia) and 2 mL of 125 mg/mL Dihydrostreptomycin (Sigma, St Louis, MO, United States) at surgery and for 3 days following surgery. Fetuses received an intramuscular injection of 1 mL of Duplocillin (150 mg/mL procaine penicillin and 112.5 mg/mL benzathine penicillin); and 1 mL of 125 mg/mL Dihydrostreptomycin during surgery. All ewes received meloxicam (0.5 mg/kg, subcutaneously) for analgesia on the day before surgery and 24 h later (48). Each fetus received antibiotics (500 mg; sodium ampicillin, Commonwealth Serum Laboratories) intra-amniotically for 4 days post-surgery.

### 2.4 Experimental protocol

Pregnant ewes underwent MRI scans between 116–119 days GA. Ewes were fasted for at least 12 h before MRI. General anaesthesia was induced in the ewe with intravenous diazepam (0.3 mg/kg) and ketamine (5 mg/kg) and maintained with 2–2.5% isoflurane (Lyppards, South Australia, Australia). The ewe was then positioned on its left side for the duration of the scan and ventilated to create normal fetal oxygen levels (respiratory rate 16–18; ~1 L O_2_ and 5 L air). Maternal heart rate and arterial oxygen saturation were measured using an MRI compatible SaO_2_/heart rate monitor (Nonin Medical Inc., Plymouth, United States). The sensor was placed on the pregnant ewe’s teat and measurements were continuously recorded using LabChart 7 (31, 51).

The fetal femoral artery and amniotic catheters were connected to displacement transducers, a quad-bridge amplifier, and a data acquisition unit (PowerLab, ADInstruments, Castle Hill, Australia) to record fetal blood pressure (corrected for amniotic pressure). All data were sampled at a rate of 1,000 Hz, digitized, and recorded using LabChart 7 (ADInstruments, Castle Hill, Australia). The resulting fetal blood pressure signal acted as a real time cardiac trigger for fetal MRI scanning and the maternal heart rate from the sensor was placed on the pregnant ewe’s teat was used as the gating signal for the uterine arteries (7, 52, 53).

Imaging was performed on a 3 Tesla clinical MRI system (MAGNETOM Skyra, Siemens Healthineers, Erlangen, Germany). Fetal vessel blood flow measurements and oxygen saturations were determined by PC and T_2_ relaxometry MRI techniques, respectively, as previously described (31, 38, 54). MRI measurements were taken firstly in a normoxaemic state, in which the oxygen supply to the ewe was titrated to match fetal blood gas status as determined in the morning of the MRI day whilst the ewe was conscious (both controls and FGR). The MRI measurements were then repeated in a state of acute hypoxaemia by reducing maternal SpO_2_ to ~80–85% via a reduction of oxygen and/or the addition of nitrogen into the inspired air. MRI acquisitions during the acute hypoxaemia period began 10 min after maternal SpO_2_ was reduced and stable.

### 2.5 Determination of blood flow within the uterine arteries and the fetal circulation

The fetal femoral arterial pressure waveform was used to generate a cardiac trigger for MR imaging (52, 53). Two-dimensional cine PC imaging was performed to measure blood flow within the fetal circulation with corresponding vessel appropriate velocity encoding (VENC). PC-MRI acquisitions were completed for the ascending aorta (AAo; VENC = 150 cm/s), main pulmonary artery (MPA; 150 cm/s), descending aorta (DAo; 150 cm/s), superior vena cava (SVC; 100 cm/s), ductus arteriosus (DA; 150 cm/s), left and right pulmonary arteries (LPA/RPA; 80 cm/s), left and right carotid arteries (CCA; 100 cm/s), umbilical vein (UV; 50 cm/s) and ductus venosus (DV; 100 cm/s) using the following parameters: flip angle: 30°; repetition time (TR): 5.6 ms; echo time (TE): 3.18 ms; field of view (FOV): 240 mm; in-plane resolution: 1.0 × 1.0 mm^2^; slice thickness: 5.0 mm (i.e., voxel size: 1.0 × 1.0 × 5.0); number of signal averages: 3; views per segment: 2; acceleration factor: 2, according to our previously published technique (31, 40, 55). With 15 acquired phases in the cardiac cycle, these parameters achieve a temporal resolution of ~28 ms. The typical acquisition time for each vessel was ~2–3 min. PC cine images were acquired in the short axis plane of the vessels of interest, which were prescribed using two perpendicular long axis views of each vessel. Pulmonary blood flow (PBF) was determined as the sum of blood flow in the LPA and RPA. Right ventricular cardiac output (RVCO) was determined as the sum of DA and PBF. Left ventricular cardiac output (LVCO) was determined as equal to AAo blood flow and did not include coronary blood flow. Combined ventricular output was determined as sum of RVCO and LVCO. During the normoxia state, blood flow was measured in the uterine arteries as previously described (7, 51).

### 2.6 Determination of oxygen saturation within the fetal circulation

Due to the paramagnetic properties of deoxyhaemoglobin, the T_2_ relaxation time of blood is related to the oxygen saturation of blood (56). Vessel T_2_ oximetry was performed using a T_2_-prepared pulse sequence with a balanced steady-state free precession acquisition (Myomaps, Siemens) (20, 44, 54, 57). MRI acquisition parameters over all subjects and vessels were: in-plane resolution: 1.3 × 1.3 mm; slice thickness: 6 mm; TR: 4.2 ms, TE: 2.1 ms, FOV: 350 mm, acceleration factor: 2, flip angle: 70°, T2 preparation times: 32, 64, 96, 128, 160, 192 ms, and acquisition time: ~50–60 s. A non-rigid motion correction algorithm was applied to compensate for slight in-plane fetal movement (co-registration) (58).

The T_2_ relaxation time for each vessel of interest was analysed using a custom lab-developed software written in Python (https://github.com/shportnoy/blood_roi_tool). The regions-of-interest were manually adjusted for each image slice to cover the central 60% of the vessel of interest (UV, DV, AAo, MPA, DA, DAo & SVC) (34, 59). Oxygen saturation was then calculated from T_2_ relaxation time using the T_2_-oxygen saturation relationship for sheep blood as previously described (54).

### 2.7 Determination of oxygen delivery and consumption

Blood flow and T_2_ derived oxygen saturations were combined to calculate overall fetal DO_2_, fetal VO_2_, cerebral DO_2_, cerebral VO_2_ and pulmonary DO_2_ using the following equations:

1. Fetal DO_2_:
$${\mathrm{DO}}_{2}=1.36\times \left[Hb\right]\times Y_{UV}\times Q_{UV}$$
2. Cerebral DO_2_:
$${\mathrm{DO}}_{2}=1.36\times \left[Hb\right]\times Y_{AAo}\times Q_{CCa}$$
3. Fetal VO_2_:
$${VO}_{2}=1.36\times \left[Hb\right]\times \left(Y_{UV}-Y_{DAo}\right)\times Q_{UV}$$
4. Cerebral VO_2_:
$${VO}_{2}=1.36\times \left[Hb\right]\times \left(Y_{AAo}-Y_{SVC}\right)\times Q_{CCa}$$

where Q_UV_ represents the measured umbilical vein blood flow; Q_CCa_ represents the combined blood flow of the left and right carotid arteries; [Hb] represents the mean fetal haemoglobin concentration during MRI scan measured using fetal arterial blood sampling and conventional blood gas analysis; 1.36 is the amount of oxygen (mL at 1 atmosphere) bound per gram of haemoglobin; Y_UV_ represents the oxygen saturation of UV blood; Y_DAo_ represents the oxygen saturation of the DAo blood, Y_AAo_ represents the oxygen saturation of AAo blood and Y_SVC_ represents the oxygen saturation of the SVC.

### 2.8 Determination of fetal and brain weight from MRI volumetry

A three-dimensional steady-state free procession of the uterus (*T*_E_ = 1.45 ms; repetition time (TR) = 3.38 ms; flip angle: 35 deg; in-plane resolution: 1.5 × 1.5 mm; slice thickness = 2 mm; number of slices = 100–120; number of averages = 1; base resolution: 272; FOV = 400 mm; average acquisition time 4–5 min) was acquired and segmented using ITK-SNAP (version 3.8 (60)) to measure fetal and brain volumes (39). Fetal and brain volumes were used to estimate fetal and brain weights using previously described tissue specific conversion factors (61, 62).

### 2.9 Blood sampling and fetal blood gas measurements

After fetal surgery, fetal arterial blood samples were collected daily to monitor fetal health by measuring the partial pressure of oxygen (PaO_2_), partial pressure of carbon dioxide (PaCO_2_), oxygen saturation (SaO_2_), pH, haemoglobin, haematocrit, base excess and lactate, temperature corrected to 39°C for sheep blood with a RAPIDPOINT 500 (Siemens Healthineers, Erlangen, Germany). During the MRI scan, arterial samples (0.5 mL) for fetal blood gas analysis were taken at the beginning and end of each state (normoxia and acute hypoxia).

### 2.10 Statistical analysis

Data were determined to be normally distributed using the Shapiro–Wilk test for normality. To determine the impact of fetal group (control vs. FGR), oxygenation state (normoxia vs. acute hypoxia) and their interaction, data were analysed by a repeated measures two-way ANOVA with a Bonferroni correction for multiple comparisons (GraphPad Prism version 8 for Windows, GraphPad Software, La Jolla California United States). Data are presented as mean ± SD and a probability of 5% (*p* < 0.05) was considered significant for all analyses.

## 3 Results

### 3.1 Uterine artery blood flow and fetal characteristics

**Figure 1:**
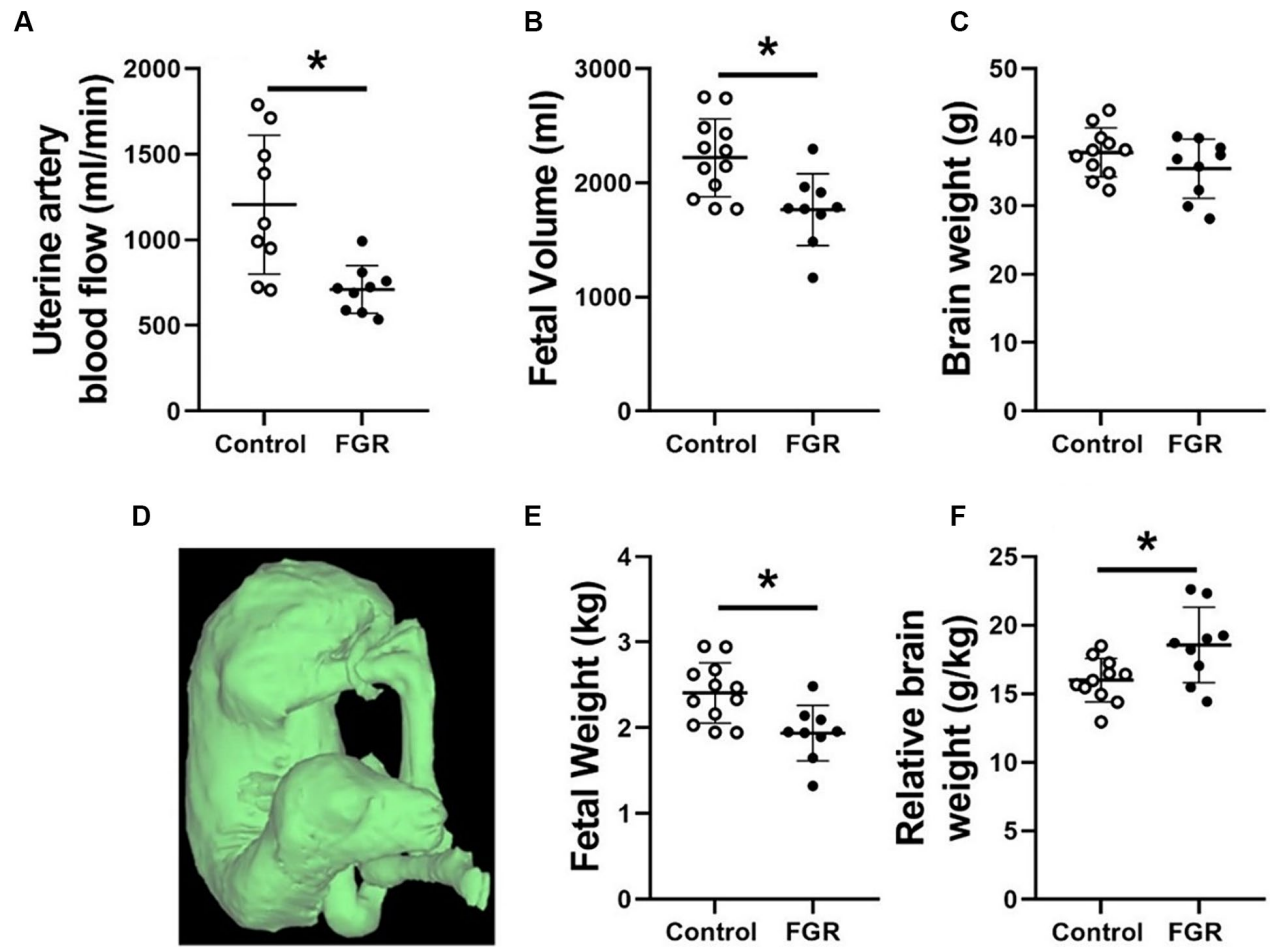
Maternal uterine artery blood flow **(A)**, fetal volume **(B)**, calculated brain weight **(C)**, representative fetal volume segmentation **(D)**, calculated fetal weight **(E)** and brain weight relative to fetal weight **(F)** in normally grown control (unfilled circles) and FGR (filled circles) fetuses. Data are presented as individual data points with mean ± SD superimposed. Data analysed using a student’s unpaired *t*-test. ^*^Statistically significant difference between groups. *p* < 0.05.

Uterine artery blood flow was significantly lower in pregnant ewes carrying a growth restricted fetus (Figure 1A). The FGR group had significantly lower fetal volume than controls (Figures 1B,D) and when converted to weight, this also translated to the FGR group being significantly smaller than appropriately grown controls (Figure 1E). Fetal brain weight was similar between groups (Figure 1C); however, the FGR group had an increased relative brain weight when normalised to body weight (Figure 1F).

Prior to MRI and during the normoxia MRI acquisition state, growth restricted fetuses had significantly lower PaO_2_ and SaO_2_ than controls (Table 1). PaO_2_ and SaO_2_ were significantly lower during the hypoxia state than both the normoxia state and prior to MRI in both groups. There was no difference in PaO_2_ and SaO_2_ between FGR and controls during the hypoxia state, indicating that similar levels of fetal hypoxaemia were achieved. pH was not different between groups prior to MRI or during either normoxia or hypoxia states. pH was significantly lower in both fetal groups during the normoxia and hypoxia states in comparison to before MRI. Lactate was not different between fetal groups prior to MRI, during normoxia or hypoxia states. Lactate was higher in both fetal groups in comparison to before the MRI in the normoxia state and lactate was significantly higher during the hypoxia state in comparison to the normoxia state (Table 1). Hb and Hct were not different between control and FGR groups but were lower during both MRI states in comparison to before MRI. PaCO_2_ was not different between fetal groups and did not change during either MRI state in comparison to before the MRI (Table 1).

**Table 1:** Mean gestational fetal blood gas, Hb and lactate values.

	Morning of MRI		Normoxia		Hypoxia		*p*-values		
	Control (*n* = 12)	FGR (*n* = 9)	Control (*n* = 12)	FGR (*n* = 9)	Control (*n* = 12)	FGR (*n* = 9)	Group	State	Group × state
*Maternal parameters*									
Maternal SPO_2_ (%)	—	—	97 ± 2	95 ± 5	87 ± 4^¥^	88 ± 6^¥^	0.7249	***<0.0001***	***0.0180***
Maternal heart rate (bpm)	—	—	92 ± 12	90 ± 6	97 ± 12	91 ± 7	0.4108	0.1966	0.2963
*Fetal arterial parameters*									
PaO_2_ (mmHg)	19.9 ± 2	16.3 ± 2.4^*^	20.6 ± 1.8	16.2 ± 2.9 ^*^	12.6 ± 2.5^#¥^	12.1 ± 1.7^#¥^	***0.0015***	***<0.0001***	***0.0079***
PaCo_2_ (mmHg)	49.7 ± 4.3	51.6 ± 7.7	55.5 ± 11.6	57.3 ± 6.3	50.7 ± 12.1	55.6 ± 7.2	0.4046	0.0551	0.7054
pH	7.337 ± 0.023	7.372 ± 0.028	7.270 ± 0.050^#^	7.277 ± 0.030^#^	7.273 ± 0.053^#^	7.277 ± 0.041^#^	0.8979	***<0.0001***	0.7272
SaO_2_ (%)	62.6 ± 5.8	49.2 ± 8.1^*^	58.5 ± 7.5	47.4 ± 7.3^*^	29 ± 7.6^#¥^	27.4 ± 6.1^#¥^	***0.0024***	***<0.0001***	*0.0525*
Hb (g/L)	128 ± 15	127 ± 13	98 ± 9^#^	92 ± 10^#^	98 ± 11^#^	100 ± 10^#^	0.2556	**<0.0001**	0.2561
Hct (%)	37 ± 5	36 ± 4	29 ± 3	25 ± 4	29 ± 3	27 ± 4	0.0923	**<0.0001**	0.2728
Lactate (mmol/L)	1.28 ± 0.33	1.32 ± 0.18	2.68 ± 1.37^#^	2.75 ± 1.32^#^	3.54 ± 1.68^#^	3.81 ± 1.80^#¥^	0.9827	***<0.0001***	0.6645

Values are mean ± SD. Data analysed by a repeated measures two-way ANOVA with Bonferroni’s correction for multiple comparisons. ^#^Significantly different to conscious state. ^¥^Significantly different to normoxia state. ^*^Significantly different between control and FGR groups within a given state. Bolded italic values are those values that represent statistical significance (*p* < 0.05).

### 3.2 Impact of acute hypoxia and FGR on fetal blood pressure and heart rate

There was no significant difference in heart rate, SBP, DBP or MAP between control and FGR groups during the MRI (Table 2). Heart rate, SBP, DBP and MAP were not significantly different between normoxia and acute hypoxia MRI acquisition states (Table 2).

**Table 2:** Fetal heart rate and blood pressure during MRI in normoxia and acute hypoxia states.

	Normoxia		Acute hypoxia		*p*-values		
	Control (*n* = 12)	FGR (*n* = 9)	Control (*n* = 12)	FGR (*n* = 9)	Group	State	Group × state
Heart Rate (bpm)	140 ± 10	142 ± 16	146 ± 21	148 ± 19	0.3869	0.3090	0.3438
SBP (mmHg)	42 ± 5	44 ± 9	45 ± 6	45 ± 8	0.8513	0.1684	0.6201
DBP (mmHg)	27 ± 4	27 ± 5	29 ± 6	28 ± 7	0.0733	1.2680	0.9384
MAP (mmHg)	35 ± 7	34 ± 6	37 ± 9	34 ± 5	0.5419	0.1070	0.1446

Values are means ± SD. Data analysed by a repeated measures two-way ANOVA with a Bonferroni correction for multiple comparisons. ^*^Significant difference between control and FGR groups (*p* < 0.05). ^#^Significantly different to conscious state. ^¥^Significantly different to normoxia state. SBP, systolic blood pressure; DBP, diastolic blood pressure; MAP, mean arterial pressure.

### 3.3 Impact of FGR and acute hypoxia on UV, DV and FO blood flow

**Figure 2:**
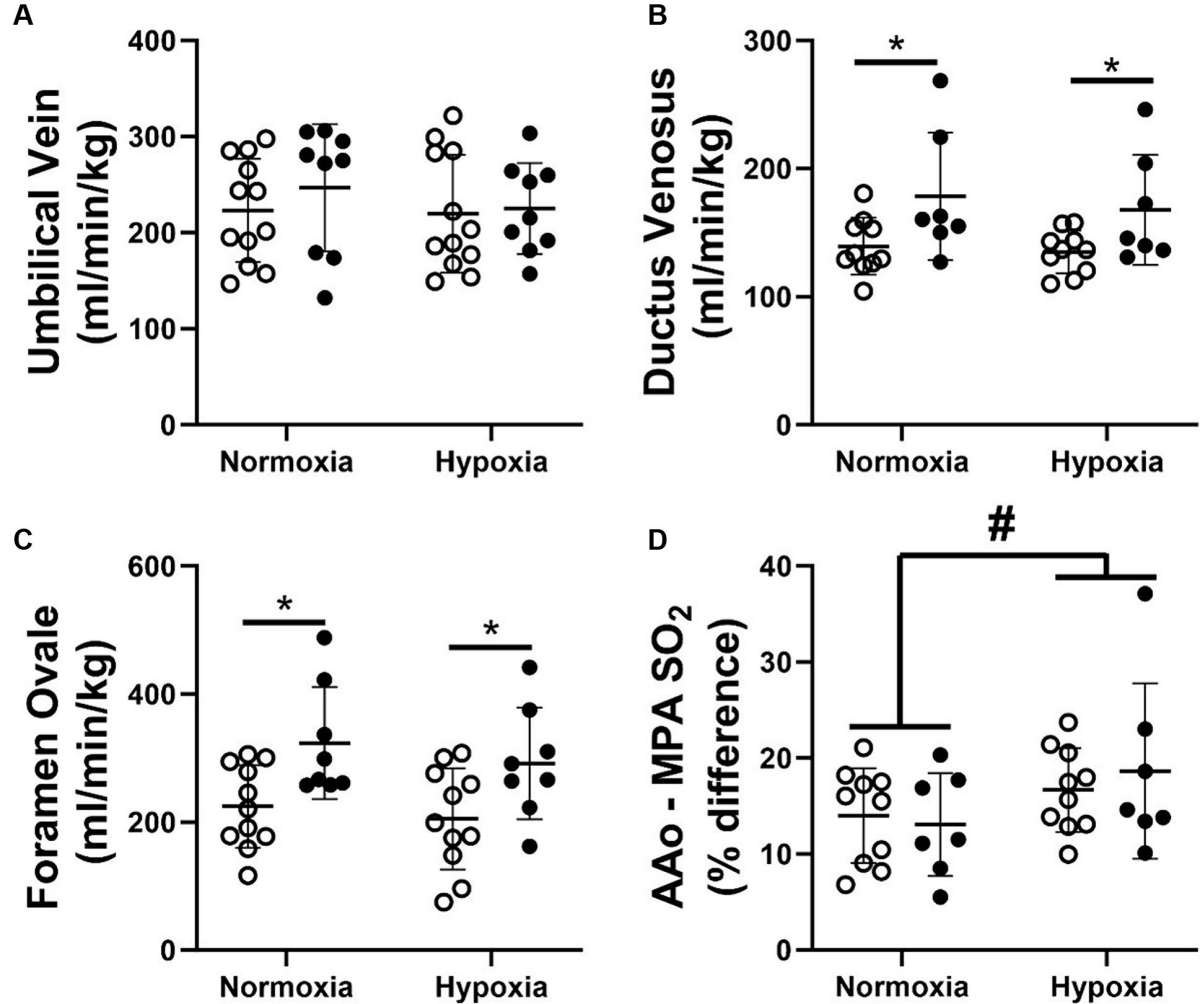
Blood flow through the umbilical vein **(A)**, ductus venosus **(B)**, foramen ovale **(C)** and the difference in blood oxygen saturation between the left and right ventricles **(D)** of normally grown control (unfilled circles) and FGR (filled circles) fetuses during nomoxia and hypoxia states. Data are presented as individual data points with mean ± SD superimposed. Data analysed by a repeated measures two-way ANOVA with a Bonferroni correction. ^*^Statistically significant difference between groups. ^#^Significant difference between normoxia and hypoxia states. *p* < 0.05.

There was no impact of FGR or acute hypoxia on blood flow through the UV (Figure 2A). However, blood flow through the DV and FO was significantly increased due to FGR during both the normoxia and acute hypoxia oxygenation states (Figures 2B,C, respectively). Despite being chronically hypoxaemic, FGR fetuses exhibited a similar blood SO_2_ difference between the AAo and the MPA (Figure 2D). This blood SO_2_ difference increased in magnitude in both control and FGR fetuses during the acute hypoxia state.

### 3.4 Impact of FGR and acute hypoxia on blood flow within the fetal circulation

**Figure 3:**
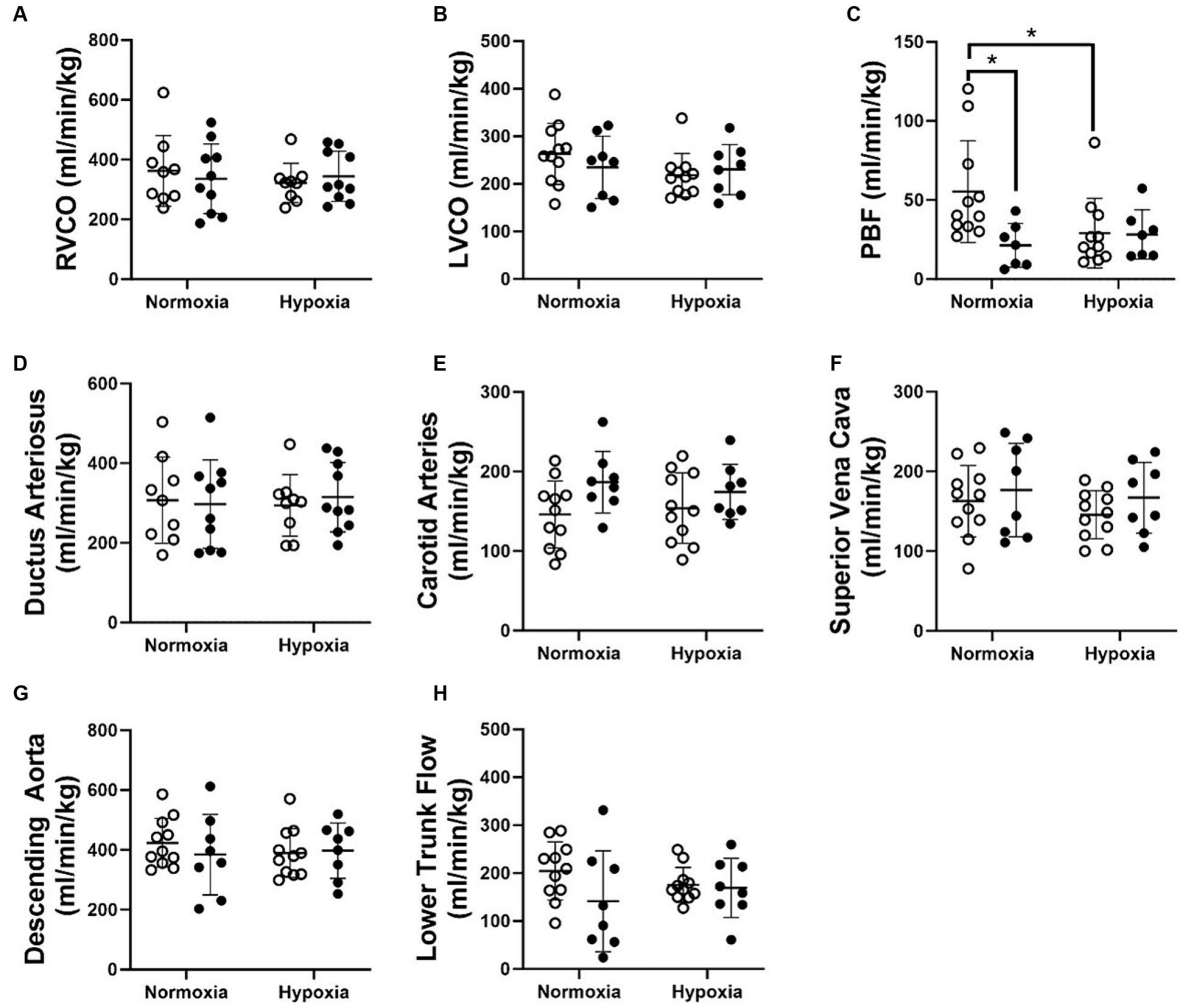
RVCO **(A)**, LVCO **(B)**, PBF **(C)** as well as blood flow through the ductus arteriosus **(D)**, carotid arteries **(E)**, superior vena cava **(F)**, descending aorta **(G)** and towards the lower trunk **(H)** in normally grown control (unfilled circles) and FGR (filled circles) fetuses. Data are presented as individual data points with mean ± SD superimposed. Data analysed using a repeated measures two-way ANOVA with a Bonferroni correction. ^*^Statistically significant difference between groups. *p* < 0.05.

There was no impact of FGR or oxygenation state on RVCO (Figure 3A). FGR decreased pulmonary blood flow (PBF) in comparison to normally grown control fetuses under normoxic conditions. However, there was no difference in PBF between normally grown control and FGR fetuses during acute hypoxia (Figure 3C). The blood flow passing through the DA was not impacted by either FGR or acute hypoxia (Figure 3D). Neither FGR nor oxygenation status had an impact on LVCO (Figure 3B) or the blood flowing toward the brain via the carotid arteries (Figure 3E). There was no impact of FGR or oxygenation state on the blood flow through the superior vena cava (Figure 3F), descending aorta (Figure 3G) or the blood flowing toward the lower trunk of the fetus (Figure 3H).

**Figure 4:**
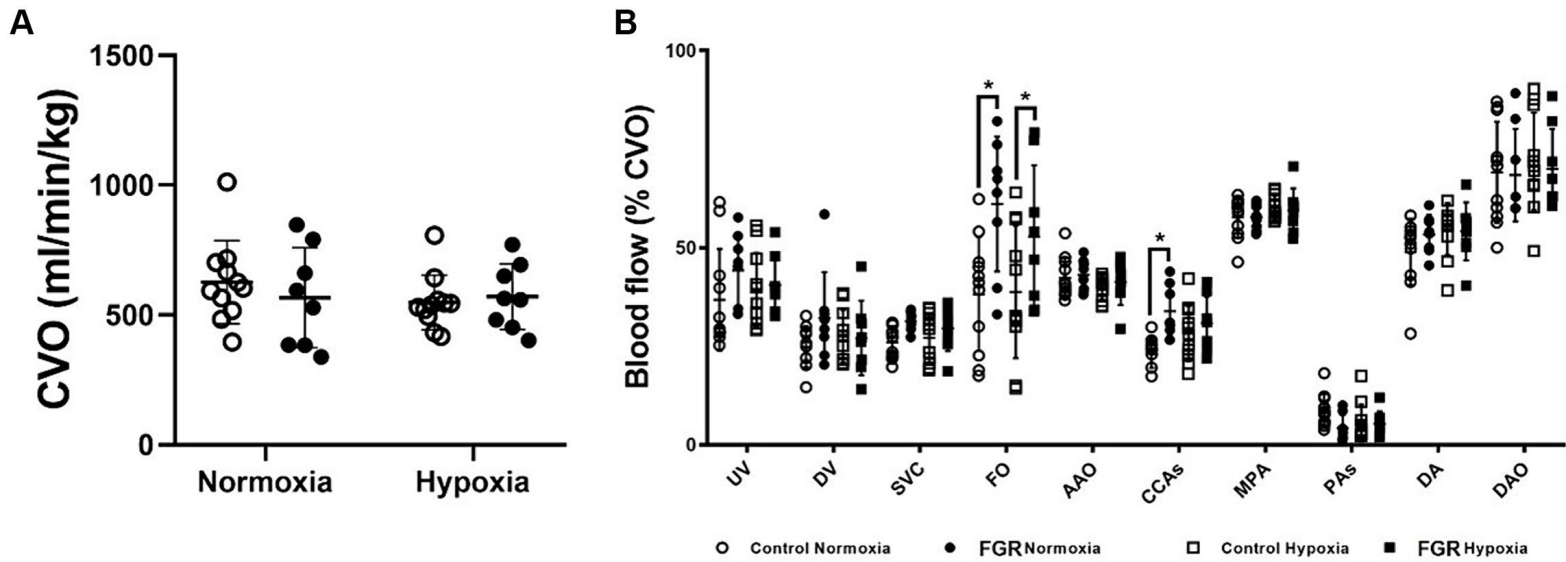
Effect of FGR and acute hypoxia on combined ventricular output (CVO; **A**) and blood flow distribution as a percentage of CVO within the fetal circulation **(B)**. Data are presented as individual data points with mean ± SD superimposed. Data analysed using a repeated measures two-way ANOVA with a Bonferroni correction. ^*^Statistically significant difference between groups. *p* < 0.05.

**Figure 5:**
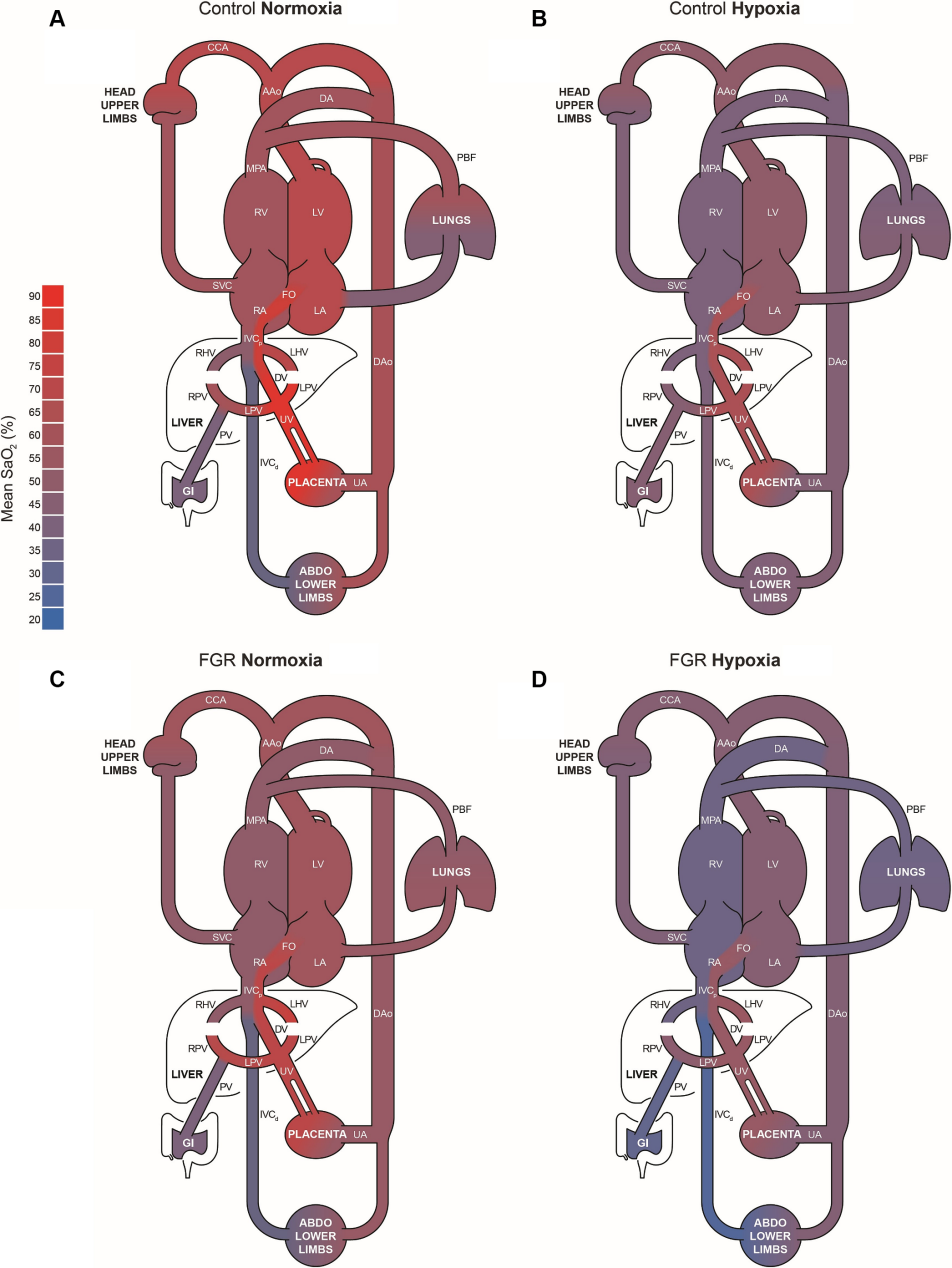
Colour coded diagrammatic representations of oxygen transport throughout the fetal circulation in control **(A, B)** and FGR **(C, D)** fetuses during normoxia and hypoxia states. Differences in the width of each vessel indicate where there are significant differences in blood flow between states and across groups. AAo, ascending aorta; CCA, combined carotid arteries; DA, ductus arteriosus; DAo, descending aorta; DV, ductus venosus; FO, foramen ovale; IVC, inferior vena cava; LA, left atrium; LHV, left hepatic vein; LV, left ventricle; MPA, main pulmonary artery; PBF, pulmonary blood flow; PV, portal vein; RA, right atrium; RV, right ventricle; RHV, right hepatic vein; SVC, superior vena cava; UA, umbilical artery; UV, umbilical vein.

There was no impact of FGR or acute hypoxia on combined ventricular output (CVO; Figure 4A). Blood flow distribution as a % of CVO was altered such that there was an increased proportion of blood flow through the FO in FGR fetuses during both the normoxia and acute hypoxia states. Blood flow distribution toward the brain through the carotid arteries (CCa) was higher in FGR than control fetuses during the normoxia state but not different during the acute hypoxia state (Figure 4B). Diagrammatic representations of oxygen transport throughout the fetal circulation in control and FGR fetuses during normoxia and acute hypoxia states are shown in Figure 5.

### 3.5 Impact of FGR and acute hypoxia on DO_2_ and VO_2_

**Figure 6:**
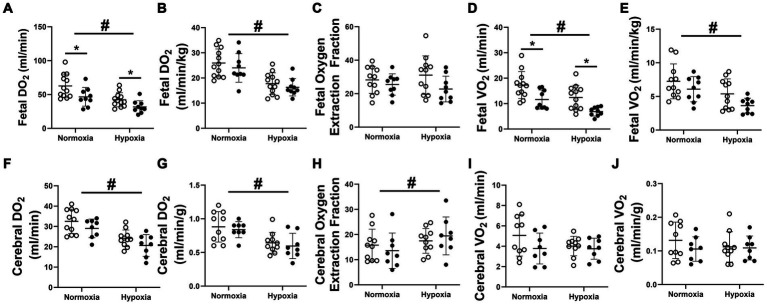
Fetal absolute **(A)** and normalised **(B)** DO_2_, oxygen extraction fraction **(C)** and absolute **(D)** and normalised **(E)** VO_2_ as well as absolute **(F)** and normalised **(G)** cerebral DO_2_, cerebral oxygen extraction fraction **(H)** and absolute **(I)** and normalised **(J)** cerebral VO_2_ in normally grown control (unfilled circles) and FGR (filled circles) fetuses. Data are presented as individual data points with mean ± SD superimposed. Data analysed by a repeated measures two-way ANOVA with a Bonferroni correction. ^*^Significant difference between normally grown and growth restricted fetuses. ^#^Statistically significant difference between oxygen states. *p* < 0.05.

Absolute fetal DO_2_ (Figure 6A) and VO_2_ (Figure 6D) of growth restricted fetuses were significantly lower than normally grown control fetuses in both normoxia and hypoxia states. Absolute fetal DO_2_ and VO_2_ of both FGR and control fetuses were significantly lower in the hypoxia state in comparison to the normoxia state. When normalised to fetal weight, fetal DO_2_ and VO_2_ were similar between control and FGR fetuses during both nomoxia and hypoxia states but significantly decreased in both groups during the hypoxia state (Figures 6B,E). There was no impact of FGR or oxygenation state on overall fetal oxygen extraction fraction (Figure 6C). FGR did not impact absolute or normalised cerebral DO_2_ and VO_2_. In line with the decreased fetal DO_2_ during the hypoxia state, both absolute (Figure 6F) and normalised (Figure 6G) cerebral DO_2_ in both normally grown and FGR fetuses was significantly decreased. Both normally grown and growth restricted fetuses had a significant increase in cerebral oxygen extraction fraction (Figure 6H) during the hypoxia state and an unchanged absolute (Figure 6I) and normalised (Figure 6J) cerebral VO_2_ compared to the normoxia state.

## 4 Discussion

In this study, we aimed to comprehensively characterize and differentiate the haemodynamic responses occurring during acute and chronic periods of fetal hypoxaemia. Fetal hypoxaemia may present as either an acute event or a chronic condition, both of which are associated with poor short-term outcomes during fetal life, such as FGR and stillbirth. These conditions can also result in impaired fetal development and developmental programming, leading to an increased risk of complications during the neonatal period and long-term morbidity across the individual’s life course. The ability to distinguish between these two scenarios and understanding their impact on fetal haemodynamics is required for development of appropriate diagnostic tools and intervention strategies. Herein, we employed a well-established pregnant sheep model of early onset placental insufficiency (19, 63, 64), alongside clinically relevant advanced MRI techniques (31, 43, 54, 65, 66), enabling exploration of the intricate relationship between fetal haemodynamics and acute versus chronic fetal hypoxaemia.

Pregnant ewes in the FGR group of this study had significantly lower uterine artery blood flow than ewes in the control group. Thus, in combination with poor placental function, this translated to chronic fetal hypoxaemia as evidenced by significantly lower PaO_2_ and SaO_2_ levels in FGR compared to normally grown fetuses. Importantly, these fetuses were already smaller than the normally grown fetuses of the control group at the time of MRI assessment, with an increased brain to body weight ratio, indicating that haemodynamic adaptations associated with “brain sparing” physiology were already in place (15, 19, 21, 67, 68). It is well established that blood flow and oxygen delivery throughout the fetal circulation are sensitive to the prevailing oxygen availability (15). As such, to best recapitulate each fetuses’ normal circulation prior to maternal ventilation, the level of maternal inspired oxygen during the normoxic MRI state was titrated on a case-by-case basis to match fetal blood gas status to that during the conscious state prior to the MRI. This approach resulted in lower PaO_2_ and SaO_2_ in FGR fetuses and is consistent with the measurement of reduced fetal DO_2_ and VO_2_ as determined by MRI. However, it should be noted that when fetal DO_2_ and VO_2_ were normalized to fetal weight, the significant difference in these measures between the control and growth-restricted fetuses was mitigated. This is in contrast to ultrasound and MRI based studies in humans, which found the significant reduction in fetal DO_2_ within the FGR group is maintained after the normalization for fetal weight (20, 69). This may be explained either by a species specific aspect or differences in the timing, duration and severity of the growth restriction induced in the present study compared to the aforementioned human studies that focused on late-onset FGR (14, 20, 68).

Similar to the MRI based study in late-onset human FGR cases by Zhu et al. (20), we comprehensively characterised the impact of chronic hypoxaemia on the major vessels within the fetal circulation in a model of early-onset FGR. Uniquely, the fetal circulation incorporates specialised shunts: the foramen ovale and ductus arteriosus, that enable blood to bypass the fetal lungs (70). An additional fetal shunt, the DV, redirects a portion of the well oxygenated umbilical venous return away from the liver and toward the posterior side of the inferior vena cava where it is preferentially streamed toward the foramen ovale and shunted from the right to the left side of the heart (52, 54, 71, 72). In the present study, we found that chronically hypoxaemic FGR fetuses had increased blood flow through the DV. This finding builds on our previous work using MRI to characterise the fetal circulation of late-onset growth restricted human fetuses, where we did not measure blood flow through the DV (20), and is consistent with the seminal ultrasound based studies evaluating the umbilical circulation in FGR human fetuses with and without abnormal umbilical artery (UA) pulsatility index (73–75). This study adds to our knowledge of the fetal circulation in the setting of growth restriction by showing that, in addition to increased DV flow, right to left heart shunting through the FO was increased with a corresponding decrease in PBF. Given that left ventricular preload blood pool is comprised of both oxygen-rich blood being shunted through the FO from the right side of the heart and pulmonary venous return, this finding suggests that the composition of the left ventricular preload blood pool in FGR fetuses is shifted to include a greater proportion of oxygen-rich blood from the DV-FO pathway. Utilising T_2_ oximetry, we confirmed this notion, and show that despite the FGR fetuses in the present study being systemically hypoxaemic, the percent difference in SaO_2_ between the blood in ascending aorta flowing towards the brain and the blood in the MPA was similar between chronically hypoxaemic FGR and control fetuses. Although this is beneficial for the brain *in utero*, the alterations in both hepatic and pulmonary haemodynamics required to aid in and ensure this process may be to the detriment of the liver and lungs, respectively. The increased DV shunting and thus reduced liver perfusion of well oxygenated and glucose rich blood returning from the placenta may contribute towards impaired liver growth in hypoxaemic growth restricted fetuses (76), a factor that is associated with reduced concentrations of key fetal growth factors, insulin-like growth factor (IGF)-1 and IGF-2 (77), and may predispose FGR born offspring to metabolic syndrome and impaired drug metabolism across the life course (67, 78–82). Moreover, hypoxaemia driven increases in pulmonary vascular resistance, and thus reduced PBF and DO_2_, may be the mechanism by which growth restricted fetuses exhibit delayed surfactant maturation (83, 84) and increased rates of persistent pulmonary hypertension (85).

Interestingly, and contrary to studies investigating haemodynamic changes during acute fetal hypoxemia that typically exhibit increased blood flow towards the brain (15, 86, 87), we found no significant difference in absolute carotid artery blood flow between control and FGR groups. Similar findings have been shown in human (20) and sheep (19, 21) studies of FGR, whereby either more than a third or the entirety of FGR fetuses studied exhibited normal cerebral blood flow. However, it should be noted that when assessed as a proportion of CVO, blood flow toward the brain was increased in FGR fetuses during the normoxic MRI acquisition state but not different to normally grown controls during the acute hypoxia state. Despite the prevailing level of fetal hypoxemia, cerebral DO_2_ in the FGR group remained similar to controls, supporting the presence of an alternative “brain sparing” mechanism in chronically hypoxaemic FGR fetuses. Thus, a preferential streaming of oxygen rich blood via the DV-FO pathway may work in tandem with an attempt to redistribute blood flow toward the brain. The identification and characterisation of this mechanism in this well-established preclinical model of FGR may allow for better diagnosis of at risk FGR fetuses without abnormal UA pulsatility indexes by using MRI analysis of blood flow and oximetry in the fetal circulation.

Chronically hypoxaemic FGR fetuses are just as likely to experience acute hypoxaemic episodes as normally grown fetuses (e.g., acute transient cord occlusions). However, this is harder to model in a preclinical setting and is therefore less well studied. Thus, the second layer of this study was to determine whether acute-on-chronic hypoxaemia induced unique haemodynamic patterns to maintain oxygen delivery to critical tissues whilst still accommodating for the challenges posed by prolonged oxygen restriction. Previously, different conclusions on this matter have been reported with either a maintenance of the acute hypoxaemia induced cardiac output redistribution in chronically hypoxaemic fetuses (22, 23, 88, 89), a sensitization of the cardiovascular response to acute hypoxaemia (22, 23) or even a blunting of this response (90, 91). However, it should be noted that these studies focussed on the immediate (within 5 min) fetal cardiovascular response to acute hypoxia, whereas the present study aimed to characterize the haemodynamic profile of fetuses after the initial response; at which point acute hypoxaemia was established (over an hour). During the acute hypoxaemia state, both normally grown control and chronically hypoxaemic FGR fetuses reached similar levels of hypoxaemia as measured by both conventional blood gas analysis and by MRI derived fetal DO_2_. It could be argued that a limitation of the present study may be that, due to the prevailing extent of hypoxaemia in the FGR group, the magnitude of the drop in fetal oxygenation was higher in normally grown control fetuses. Although changes in fetal PaO_2_ are detected by the carotid bodies, the concept of a fixed “set point” for hypoxaemia detection by these carotid bodies and the consequent activation of a haemodynamic response is not yet fully established. The PaO_2_ achieved during the acute hypoxaemia MRI acquisition state are in line with previous studies investigating the haemodynamic responses to acute hypoxaemia in fetal sheep (86, 92). Much like advancing gestation, prior exposure to hypoxaemia may alter carotid body sensitivity and the subsequent initiation of a haemodynamic response. Therefore, different severities of decreased PaO_2_ may elicit varying responses, rather than the response being based on passing a fixed “set point.” Indeed, previous work has shown that the chronically hypoxaemic fetus regards the hypoxaemia that it operates under as normal with no changes in carotid body signaling that are seen in response to acute hypoxia (93). Although we have previously shown the chronically hypoxaemic fetus to have a greater reliance on α-adrenergic stimuli to regulate its blood pressure (94), comparative studies assessing the blood pressure responses in the presence of either a post-ganglionic or an α-adrenergic antagonist indicate that the increased reliance on α-adrenergic stimuli is due to hyperinnervation of the peripheral vasculature that is driven by hypoxaemia (95, 96). However, this hyperinnervation is not present at the gestational ages assessed in present study but rather develops as gestation progresses becoming apparent 2 weeks later at ∼130 days gestation. Reducing fetal PaO_2_ to a comparable degree in control and FGR fetuses has allowed the present study to disentangle any influence that prior and prevailing chronic hypoxaemia may have on the haemodynamic response. Overall, both control and FGR fetuses exhibited similar reductions in fetal VO_2_, a well-known fetal response to acute hypoxia often linked to reduced fetal breathing movements (97), reduced hind limb VO_2_ (98) and a general shift away from processes that may require more oxygen (15, 92).

Whilst the increased flow through the DV-FO pathway in the FGR group was maintained during the second hit of acute hypoxaemia and the blood SO_2_ percent difference between the ascending aorta and the main pulmonary artery higher than during the normoxia state, this increased right to left heart shunting was unable to maintain cerebral DO_2_. Indeed, cerebral DO_2_ in both groups was significantly lower during the acute hypoxaemia state and FGR fetuses responded identically to control fetuses, by increasing cerebral oxygen extraction fraction to maintain cerebral VO_2_. An interesting finding was the lack of change in DV blood flow in control fetuses in response to acute hypoxaemia. Previously, it has generally been considered that in response to acute hypoxaemia, blood flow through the DV increases (99–102) with evidence that this may be due to a hypoxaemia driven α—adrenergic vasoconstriction within the liver, and thus, an increased hepatic vascular resistance allowing more blood to bypass the liver in favour of the DV (102). Thus, a possible explanation of our contrasting results concerning blood flow through the DV may be the extent of α-adrenergic innervation of vascular tone within the fetal liver at the time of the study. The reliance that the fetus has for blood pressure regulation through α-adrenergic innervation of the vasculature not only increases with chronic hypoxaemia but also with advancing gestational age (95). Although previous studies investigated the impact of acute hypoxaemia on DV flow across a range of gestational ages, to the best of our knowledge, the present study was performed at the youngest gestational age to date, and thus, this regulatory mechanism may not have been mature enough to illicit a measurable change in blood flow through the DV. Alternatively, it is possible that DV is directly rather than indirectly innervated by α-adrenergic mechanisms and that such direct innervation of the DV is not yet mature in the fetal sheep of the present study. Previously, *ex vivo* studies into the DV of late gestation fetal sheep found them to not only contain adrenergic fibres but also to respond increasingly to α-adrenergic stimuli as gestational age advanced and oxygen tension increased (103). This suggests a key interplay between the prevailing oxygen tension and gestational age in the regulation of DV tone. Indeed, placental insufficiency induced reduction in oxygen content of the blood within the UV may have altered the tone of the DV of the FGR sheep of the present study and explain the increased DV flow. Gestational age of the fetuses of the present study may also explain why we did not observe a decrease in PBF in response to acute hypoxia. Previous studies in fetal sheep have shown that the pulmonary response to hypoxaemia was not developed at 112–119 days GA (present study ~118 days GA), and that the increase in pulmonary vascular resistance and decreased pulmonary blood flow in response to acute hypoxia was only observed from 121 days GA onwards due to a progressive increase in pulmonary vasomotor tone across gestation (104). Nonetheless, we have shown that at this gestational age, increased DV-FO streaming is present in response to chronic hypoxaemia, but not yet a present adaptation in response to acute hypoxaemia.

The present study has several limitations related to the model of FGR and with the need for anaesthesia during the MRI acquisition. Herein, we utilised the well-established carunclectomy model of early onset placental insufficiency and FGR. Whilst, this model results in chronic fetal hypoxaemia, it should be noted that this is not in isolation from reductions in the supply of other substrates or hormones to the fetus (68, 94, 105, 106). As such, there is the possibility that the reductions in other metabolic substrates and/or hormones may also play a role in the fetal haemodynamic response. Although our previous work comparing the fetal haemodynamic response to acute hypoxaemia in the presence of different anaesthetic regimens found isoflurane to be the most appropriate anaesthetic agent (107); there are still caveats that need to be noted in order to fully understand our findings. The immediate haemodynamic response to acute hypoxaemia includes an initial bradycardia followed by a rise in MAP (15). However, whilst under the influence of isoflurane, there is no bradycardia but rather a short-lived tachycardia (107). Whilst our MRI acquisitions of fetal blood flow and oximetry were performed after this initial response to acute hypoxaemia had subsided and fetal heart rate was not different to the normoxia state, the flows reported may not represent the same physiological response to acute hypoxaemia as may have occurred without the presence of anaesthesia. Furthermore, isoflurane has been linked to cerebral vasodilation as well as a reduction in cerebral metabolism (108). As such, it is possible that carotid blood flow may have already been elevated above true baseline levels in the normoxia state—even before acute hypoxaemia, and thus, a significant difference in carotid artery blood flow and cerebral DO_2_ between control and FGR fetuses as well as between the normoxia and acute hypoxaemia states could have been masked.

In summary, this study has utilised a combination of clinically relevant MRI techniques and a well-established sheep model of placental insufficiency induced FGR to characterise haemodynamics of the growth restricted fetus and assess their ability to respond to a secondary episode of acute hypoxaemia. We have shown clear evidence for the importance of the preferential streaming mechanism, whereby oxygen rich blood is preferentially streamed through the DV-FO pathway to maintain cerebral DO_2_. However, this preferential streaming is aided by alterations to pulmonary haemodynamics that may manifest as respiratory complications at birth (109). When challenged with an acute episode of hypoxaemia, unlike their normally grown counterparts, PBF in FGR fetuses had already reached its lower plateau, which may have impeded further increases in FO flow to deal with the worsening hypoxaemia. The present study has laid the foundation for future studies to utilise MRI to determine how the haemodynamics in FGR change as gestation progresses, isolate the gestational age at which the DV becomes responsive to acute hypoxaemia and evaluate the maturation of the response to acute-on-chronic hypoxaemia.

## Data availability statement

The raw data supporting the conclusions of this article will be made available by the authors, without undue reservation.

## Ethics statement

The animal study was approved by Animal Ethics Committee of the South Australian Health and Medical Research Institute (SAHMRI). The study was conducted in accordance with the local legislation and institutional requirements.

## Author contributions

JRTD: Data curation, Formal analysis, Investigation, Methodology, Project administration, Software, Validation, Writing – original draft, Writing – review & editing. BSS: Data curation, Formal analysis, Investigation, Methodology, Software, Writing – review & editing. SLH: Investigation, Methodology, Project administration, Supervision, Validation, Writing – review & editing. SJH: Data curation, Formal analysis, Investigation, Software, Writing – review & editing. SRP: Data curation, Methodology, Software, Writing – review & editing. CKM: Conceptualization, Data curation, Formal analysis, Funding acquisition, Investigation, Methodology, Resources, Software, Supervision, Visualization, Writing – review & editing. MS: Conceptualization, Data curation, Formal analysis, Funding acquisition, Investigation, Methodology, Project administration, Resources, Software, Supervision, Validation, Visualization, Writing – review & editing. JLM: Conceptualization, Data curation, Formal analysis, Funding acquisition, Investigation, Methodology, Project administration, Resources, Software, Supervision, Validation, Visualization, Writing – review & editing.
